# Core components of a Community of Practice to improve community health worker performance: a qualitative study

**DOI:** 10.1186/s43058-022-00279-1

**Published:** 2022-03-10

**Authors:** Rachel Hennein, Joseph M. Ggita, Patricia Turimumahoro, Emmanuel Ochom, Amanda J. Gupta, Achilles Katamba, Mari Armstrong-Hough, J. Lucian Davis

**Affiliations:** 1 Uganda Tuberculosis Implementation Research Consortium, Makerere University, Kampala, Uganda; 2 Department of Epidemiology of Microbial Diseases, Yale School of Public Health, New Haven, CT USA; 3 Yale School of Medicine, New Haven, CT USA; 4 Clinical Epidemiology and Biostatistics Unit, Department of Medicine, Makerere University College of Health Sciences, Kampala, Uganda; 5 Department of Social and Behavioral Sciences, New York University, New York, NY USA; 6 Department of Epidemiology, New York University, New York, NY USA; 7 Pulmonary, Critical Care, and Sleep Medicine Section, Yale School of Medicine, New Haven, CT USA; 8 Center for Methods in Implementation and Prevention Science, Yale School of Public Health, New Haven, CT USA

**Keywords:** Community of practice, Behavior Change Technique, Intervention function, Low-income countries

## Abstract

**Background:**

Communities of Practice (CoPs) offer an accessible strategy for healthcare workers to improve the quality of care through knowledge sharing. However, not enough is known about which components of CoPs are core to facilitating behavior change. Therefore, we carried out a qualitative study to address these important gaps in the literature on CoPs and inform planning for an interventional study of CoPs.

**Methods:**

We organized community health workers (CHWs) from two tuberculosis (TB) clinics in Kampala, Uganda, into a CoP from February to June 2018. We conducted interviews with CoP members to understand their perceptions of how the CoP influenced delivery of TB contact investigation. Using an abductive approach, we first applied inductive codes characterizing CHWs’ perceptions of how the CoP activities affected their delivery of contact investigation. We then systematically mapped these codes into their functional categories using the Behavior Change Technique (BCT) Taxonomy and the Behavior Change Wheel framework. We triangulated all interview findings with detailed field notes.

**Results:**

All eight members of the CoP agreed to participate in the interviews. CHWs identified five CoP activities as core to improving the quality of their work: (1) individual review of feedback reports, (2) collaborative improvement meetings, (3) real-time communications among members, (4) didactic education sessions, and (5) clinic-wide staff meetings. These activities incorporated nine different BCTs and five distinct intervention functions. CHWs reported that these activities provided a venue for them to share challenges, exchange knowledge, engage in group problem solving, and benefit from social support. CHWs also explained that they felt a shared sense of ownership of the CoP, which motivated them to propose and carry out innovations. CHWs described that the CoP strengthened their social and professional identities within and outside the group, and improved their self-efficacy.

**Conclusions:**

We identified the core components and several mechanisms through which CoPs may improve CHW performance. Future studies should evaluate the importance of these mechanisms in mediating the effects of CoPs on program effectiveness.

**Supplementary Information:**

The online version contains supplementary material available at 10.1186/s43058-022-00279-1.

Contributions to the literature

- Communities of Practice may improve performance among healthcare workers through knowledge sharing, social support, and problem solving. However, additional studies are needed that identify the specific group activities and behavioral mechanisms that influence performance, in order to guide planning, implementation, and evaluation of Communities of Practice.
- We conducted a qualitative study of a Community of Practice that supported community health workers delivering contact investigation for tuberculosis in Uganda.
- By cataloging group activities and linking those activities to an established model of behavior change, we identified core components of the Community of Practice that functioned to improve community health workers’ performance.

## Background

Sub-optimal healthcare worker performance is a major barrier to delivery of high-quality health services in low- and middle-income countries [1–3]. This barrier is particularly salient for community health workers (CHWs), who have limited formal health professional education and access to training in low-income countries [4]. While many quality improvement initiatives include training to improve healthcare worker performance, a systematic review of such strategies found that training was associated with only moderate improvements in performance. When combined with group problem solving, however, large improvements were observed [3]. Furthermore, a Cochrane qualitative evidence synthesis concluded that providing continuous education and enabling CHWs to share their experiences with peers facilitated their work [5].

Communities of Practice (CoPs) offer a promising mode of delivery for continuous group learning and problem solving [6]. Communities of Practice (CoPs) are groups of people with a common work objective who meet regularly to support each other, share and create knowledge, and explore innovations [6, 7]. In their original studies among West African tailors, Lave and Wenger (1991) developed CoP theory to describe the organic learning that occurs among tradespersons and other professionals-in-training [6–8]. They theorized that the learning that happens within CoPs occurs through social interactions within the specific context where the task is meant to be performed [6, 7].

CoPs have been used to improve organizational performance within the trades, business sector, and health field [7–11]. In the trades, CoPs have been established with the goal of developing competent tradespersons through interactions between novices and experts [12, 13]. Within the business sector, CoPs have been used to improve job performance in a variety of organizations, from insurance businesses to technology firms [14–16]. Within the health field, CoPs have been established to train healthcare students, share knowledge among practicing healthcare workers, and facilitate uptake of evidence-based practices [17–32]. Empirical studies of CoPs in these sectors identified that they help participants develop their professional identities; improve their work-related knowledge, confidence, and performance; increase social capital; and enhance their social status [12, 13, 18, 21, 22, 26, 27, 29, 31–33].

Wenger (2002) describes three fundamental elements of CoPs: (1) *domain* (i.e., the subject of shared interest), (2) *community* (i.e., the social interactions and relationships among members), and (3) *practice* (i.e., the frameworks, ideas, tools, language, documents, and stories that members share) [34, 35]. Previous studies have provided empirical data that support these three characteristics. For example, studies have highlighted that consistent participation of members during CoP activities is vital to foster social interactions and build a *community* [28]. These social interactions cultivate group trust, mutual respect, and confidence to share their ideas and experiences without fear of being judged, which is critical for negotiating the group’s purpose and goals (i.e., *domain*) [14, 19, 33]. Furthermore, providing opportunities to interact with mentors and peers during and outside of work is important to develop shared frameworks, language, and tools to accomplish group goals (i.e., *practice*) [12, 13, 18, 36–38].

Despite this mounting evidence of the potential for CoPs to improve healthcare worker performance, there remain a few critical gaps in the literature pertinent to CHW CoPs in low-income countries. First, the majority of studies on CoPs have taken place in high- and middle-income countries, and additional exploration of CoP functioning in low-income countries is warranted given differences in culture, education systems, and health systems [10, 11]. Furthermore, empirical evaluations of CoPs including CHWs are sparse, as the majority focus on nurses and physicians [10, 11]. Thus, additional exploration of how CoPs function for lay healthcare workers who do not receive formalized health professional education is needed. While previous studies have focused on identifying the key elements of CoPs in healthcare, few have investigated the intervention components that elicit behavior change [10, 11, 39]. Identifying the core intervention components of CoPs (also known as the “active” components) that elicit behavior change could enable researchers and implementers to design them for continuous quality improvement [40, 41]. Because the concept and theoretical understandings of CoPs are still evolving [8, 9, 42], applying behavioral theory to data collected in empirical studies of CoPs could improve our understanding of when, where, how, and under what conditions these groups can be engineered to change behavior [39, 43]. For example, the Behavior Change Technique (BCT) Taxonomy and the Behavior Change Wheel framework provide comprehensive approaches to cataloging the core components of complex health interventions in order to design and implement strategies that optimize outcomes [44, 45].

To address these gaps in the literature, we performed a qualitative study to identify the core components of a CHW CoP formed to improve tuberculosis (TB) contact investigation in Kampala, Uganda. Through semi-structured interviews and field notes, we aimed to explore CHWs’ experiences participating in the CoP and determine the extent to which the CoP was acceptable, feasible, and effective in facilitating delivery of contact investigation. We analyzed qualitative data using the BCT Taxonomy [45] and the Behavior Change Wheel’s intervention functions [44]. In so doing, we aimed to identify behavioral mechanisms to describe how CoP activities function to improve CHW performance in low-resource settings.

## Methods

### Setting

Uganda has a high TB burden, with an annual incidence rate of 201 cases per 100,000 and an annual mortality rate of 26 deaths per 100,000 [46]. In Kampala, TB services are provided free of charge through the Uganda National TB and Leprosy Program (NTLP) and the Kampala Capital City Authority. CHWs support clinic-based health workers in delivering TB services, with funding and technical assistance from non-governmental or research organizations partnering with the NTLP. TB CHWs in Kampala receive on-the-job training specific to delivering TB services in the community and are supported through supervision by TB clinic leaders. CHWs are responsible for community-based treatment adherence support and contact investigation, as well as clinic-based TB symptom screening, education, and counseling. Clinical data is recorded in paper logbooks, or in electronic case-record forms on mobile tablets.

### CoP intervention

Our research team established a CoP in February 2018 within TB units at two public health centers in Kampala to support CHWs delivering contact investigation. The research team’s purpose for this study was to inform the implementation of CoPs in an upcoming stepped-wedge cluster randomized trial of CoPs to improve contact investigation for TB. All eight CHWs affiliated with these TB units were invited and agreed to participate in the CoP. Before the CoP began, all participating CHWs were funded through the research project, trained in contact investigation, informally communicated with each other, and received monthly supervision and feedback from TB clinic leaders. However, there was not a forum for CHWs to learn from each other or identify their own areas for improvement. Thus, the research team facilitated the establishment of a CoP to enable CHWs to problem solve, support each other, and brainstorm ways to improve contact investigation. The research team encouraged members to meet weekly on Friday mornings to share their experiences, successes, and challenges in delivering contact investigation in the prior week. The aims of these weekly meetings were to provide opportunities for members to engage with each other, develop group goals, and share stories about delivering care in the community, which relate to the three characteristics of CoPs described by Wenger (2002) (i.e., *community*, *domain*, and *practice*, respectively) [34, 35]. Leadership responsibilities rotated weekly among all participants, with a new chairperson assigned at the end of each meeting to organize and lead the discussion the following week. To catalyze discussions and develop a shared accountability structure and tool (i.e., *practice*), the research team provided performance reports listing incomplete contact investigation records for each CHW. The reports also presented facility-level process indicators for each step of the TB contact investigation cascade, including the stepwise proportions of (1) cases interviewed, (2) contacts screened, (3) eligible contacts completing evaluation, and (4) individuals diagnosed with active TB [47]. Apart from encouraging the CoP to meet weekly and providing feedback reports, the research team emphasized that the CoP should be run by its members.

### Data collection

A Ugandan, male social scientist (JG) prospectively collected field notes during weekly CoP meetings. Field notes summarized the content of meetings, participation of members, interactions between participants, and meeting tone. Research staff invited all CoP participants to interview at a place and in a language (English or Luganda) of their choice in July 2018, five months after CoP initiation. Two researchers (MAH, JG) developed an interview guide to probe how CHWs perceived their roles delivering contact investigation during CoP implementation (Additional File 1). Three Ugandan members of the research team (JG, EO, PKT) who participated in CoP implementation discussed and revised the guide after reviewing the field notes. A Ugandan, male social scientist (JG) obtained verbal consent and conducted and audio-recorded all interviews. We then de-identified and transcribed all interviews, and translated Luganda interviews into English. The interviewer (JG) revised all transcripts for accuracy. The Makerere University School of Public Health Higher Degrees Research Ethics Committee and the Yale University Human Investigation Committee approved the study. We reported all findings using the Consolidated Criteria for Reporting Qualitative Research (COREQ) checklist [48].

### Data Analysis

**Fig. 1:**
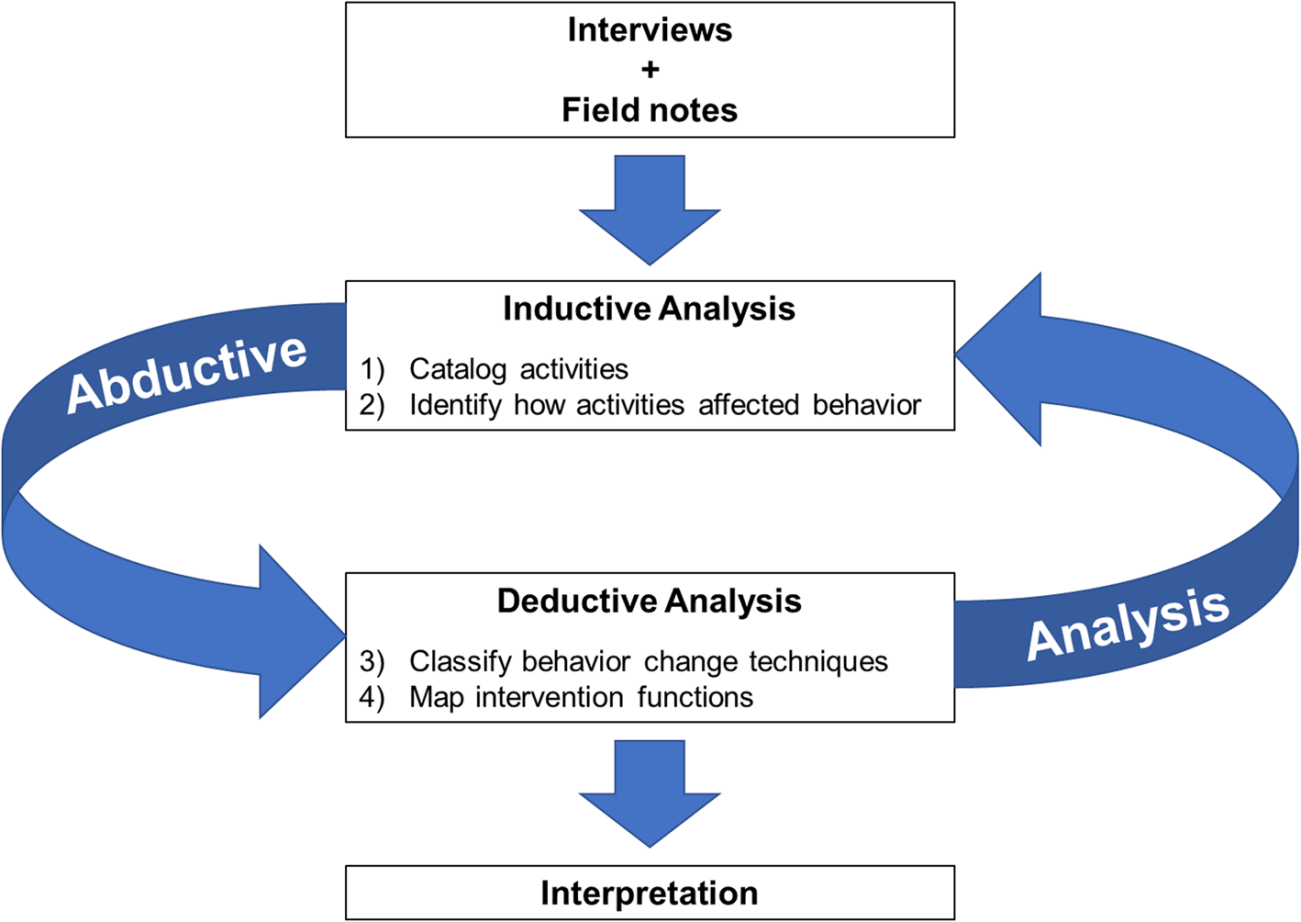
Process model for identifying behavior change techniques and intervention functions of the Community of Practice using abductive analysis

A non-Ugandan, female researcher (RH) with prior research experience in Uganda coded transcripts in ATLAS.ti using an abductive approach [49]. Abductive analysis employs both inductive codes that emerge from the data and deductive codes informed by theory. This approach facilitates conversation between empirical data and theory in order to validate, modify, and/or refute theoretical understandings of implementation [43]. We followed a four-step abductive analytic process to identify BCTs and intervention functions (Fig. 1) [50]. This included (1) cataloging activities, (2) identifying how activities affected behavior, (3) classifying BCTs, and (4) mapping intervention functions. First, the coder analyzed interviews inductively to identify CoP activities that CHWs described as benefitting their work. We cross-referenced activities that emerged from interviews with field notes and specified their actors, participants, modes of delivery, and frequencies using the Template for Intervention Description and Replication (TIDieR) checklist [51]. Second, the coder applied inductive codes to describe CHWs’ perceptions of how and why these activities influenced their delivery of TB contact investigation. In the third step, we systematically mapped inductive codes and themes to the BCT Taxonomy. The BCT Taxonomy was designed to characterize active ingredients of complex interventions [45, 52]. We adapted definitions from the BCT Taxonomy to describe the CoP. We then organized the BCTs by relevant CoP activity and noted contextual factors that facilitated CHWs’ performance. Finally, we mapped BCTs to intervention functions using the Behavior Change Wheel framework to understand underlying mechanisms through which CoPs influence practice [53]. Three authors (RH, JLD, MAH) reviewed and discussed the mapped BCTs and intervention functions to reach consensus. Ugandan team members (JG, EO, PT) and non-Ugandan researchers with extensive local research experience (JLD, MAH, AJG) reviewed and validated the code structure. We triangulated data from field notes and semi-structured interviews. We defined data saturation as the point at which novel inductive codes ceased to emerge from the data [54].

## Results

### Sample

All eight CHWs from the CoP agreed to participate in interviews. Four interviews were conducted in English, two in a mixture of English and Luganda, and two in Luganda. Interviews lasted from 48 to 69 min. Median age of participants was 39.5 years (range: 26–51) and six (75%) participants were female (Table 1). We reached data saturation after six interviews.

**Table 1:** Participant characteristics

Characteristics	***n*** (%)
Gender	
Female	6 (75)
Male	2 (25)
Age range (years)	
21–30	2 (25)
31–40	2 (25)
41–50	3 (38)
51–60	1 (12)
Education	
Vocational	1 (12)
O-Level	1 (12)
A-level	3 (38)
Diploma	1 (12)
University	2 (25)
Interview language	
English	4 (50)
Luganda	2 (25)
English and Luganda	2 (25)

Abbreviations**: ***A-level*, advanced (secondary school) level; *O-level*, ordinary (secondary school) level

### CoP content

**Fig. 2:**
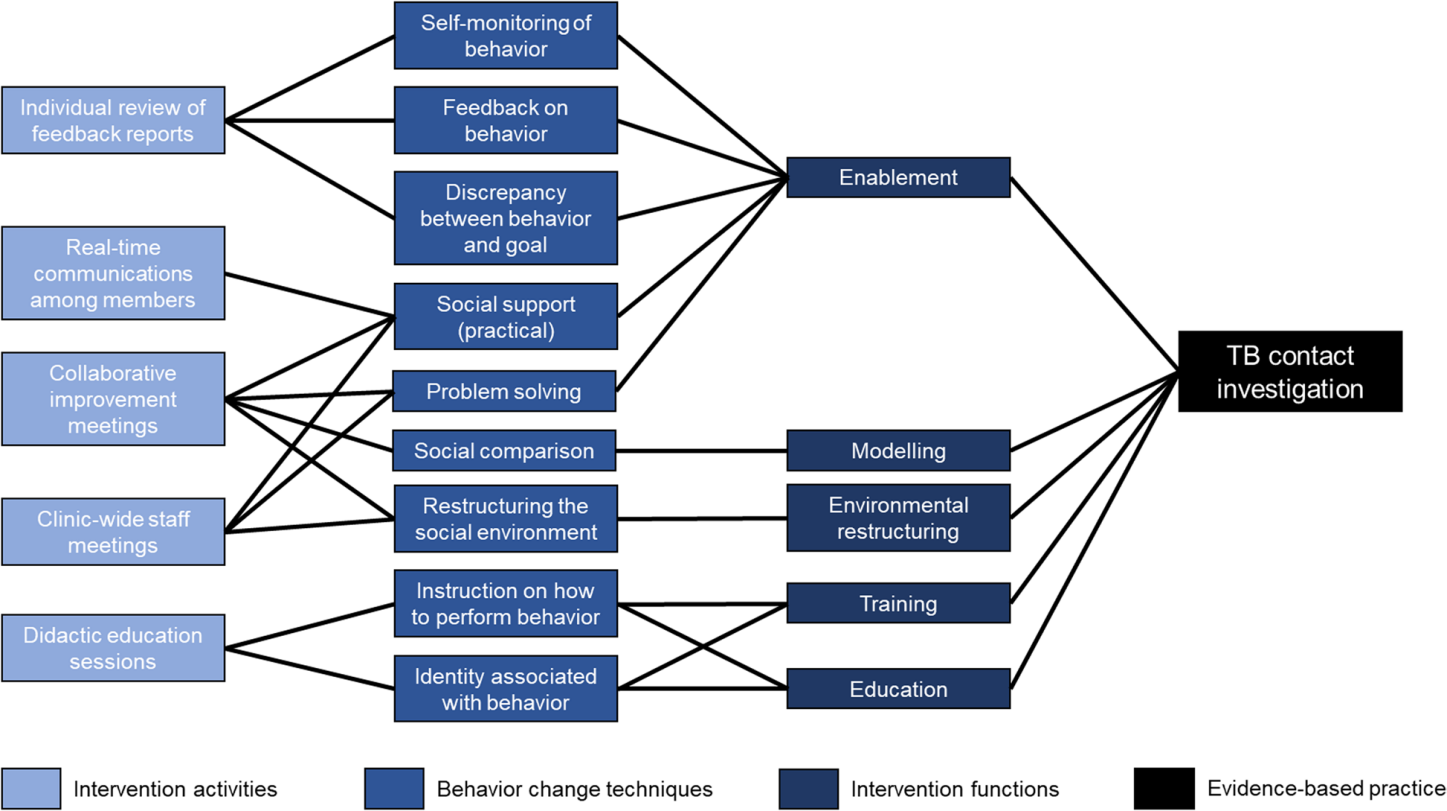
Conceptual model illustrating our implementation mapping exercise for the Community of Practice intervention. Starting at the far left, we linked intervention activities to specific behavior change techniques and related intervention functions, all to facilitate implementation of the evidence-based practice of tuberculosis contact investigation

CHWs identified five core components from the CoP that facilitated their delivery of household contact investigation services (Table 2). These included (1) individual review of feedback reports, (2) collaborative improvement meetings, (3) real-time communication among members, (4) didactic education sessions, and (5) clinic-wide staff meetings. Two of these activities were proposed by the research team to establish the CoP (i.e., review of feedback reports and collaborative improvement meetings) and three were proposed and adopted by the CoP members (i.e., real-time communications among members, didactic education sessions, and clinic-wide staff meetings). We identified relevant BCTs and Behavior Change Wheel functions for each activity discussed (Tables 3 and 4 and Fig. 2). Additional File 2 describes peripheral components that facilitated the delivery of these core components, such as cellphone data plans to enable real-time communication among members.

**Table 2:** Description of Community of Practice activities that emerged from the interviews

Description	Mode of delivery	Organized by	Participants	Frequency
*Activity 1: Individual review of feedback reports*				
Reading and reflecting on performance reports that included key indicators for the tuberculosis contact investigation cascade	Hard copy/paper; electronic copy	Research coordinator	Community of Practice members	Weekly
*Activity 2: Collaborative improvement meetings*				
Weekly meetings led by a rotating community health worker chairperson for the group to share experiences, review feedback reports with each other, and solve problems together	In-person	Community of Practice champion (champion rotated weekly)	Community of Practice members	Weekly
*Activity 3: Real-time communications among members*				
Text messages and phone calls to request immediate advice or assistance or share concerns	WhatsApp, phone calls	Community of Practice members	Community of Practice members	Continual
*Activity 4: Didactic education sessions*				
Lectures and interactive teaching sessions on topics relevant to contact investigation that were organized by the research team and delivered by invited clinicians	In-person	Community of Practice members with help from research staff and clinicians	Community of Practice members	As needed
*Activity 5: Clinic-wide staff meetings*				
Meetings with other clinic staff to provide updates on the contact investigation activities being carried out by community health workers	In-person	Community of Practice members	Clinic staff, Community of Practice members	Monthly

**Table 3:** Adapted definitions for behavior change techniques and intervention functions relevant to Community of Practice activities

Label	Adapted definition
Behavior Change Techniques	
*Self-monitoring of behavior*	Establish a method for Community of Practice members to regularly examine and record their own behavior(s) during household visits for contact investigation.
*Feedback on behavior*	Monitor and provide informative or evaluative comments to the actor on performance of contact investigation (e.g*.*, How frequently was sputum successfully collected if indicated?).
*Discrepancy between current behavior and goal*	Draw attention to differences between the Community of Practice members’ household visit metrics (i.e. contact investigation process metrics, aggregated by clinic affiliation) and the goal of completing contact investigation for all clients.
*Problem solving*	Prompt Community of Practice members to analyze factors influencing the desired outcome of completing household contact investigation and generate strategies for overcoming barriers and/or increasing facilitators.
*Social support (practical)*	Provide practical help from peers and/or supervisors to improve the performance of household contact investigation.
*Social comparison*	Draw attention to the performance of other Community of Practice members in carrying out contact investigation to emphasize similarities to and differences from each individual member’s own performance.
*Restructuring the social environment*	Change the interactions among the Community of Practice members, supervisors, and/or clinic staff to facilitate household contact investigation.
*Instruction on how to perform a behavior*	Advise or agree on how to carry out household contact investigation (includes skills training).
*Identity associated with changed behavior*	Construct a new self-perception as a more skilled community health worker conducting household contact investigation.
Intervention Functions	
*Enablement*	Increase means and/or reduce barriers for community health workers to perform contact investigation.
*Modeling*	Provide an example for community health workers to aspire to or imitate to perform contact investigation.
*Environmental restructuring*	Change the physical or social context within which community health workers deliver contact investigation services.
*Training*	Impart community health workers with skills needed to deliver contact investigation services.
*Education*	Increase community health workers’ knowledge or understanding to perform contact investigation.

Note: Definitions for Behavior Change Techniques were adapted from Michie et al. (2013) and definitions for Intervention Functions were adapted from Michie et al. (2011)

**Table 4:** Characterizing behavior change techniques and intervention functions of the Community of Practice intervention components

Intervention Components	Behavior Change Techniques	Functions
*Activity 1: Individual review of feedback reports*		
Program provided feedback reports on performance of household contact investigation (e.g., how frequently were sputum samples successfully collected if indicated) to Community of Practice members.	Feedback on behavior	Enablement
Community of Practice members reviewed their data to understand their performance in carrying out contact investigation.	Self-monitoring of behavior	Enablement
Community of Practice members viewed discrepancies between their household visit metrics and their goal of completing household contact investigation in full.	Discrepancy between current behavior and goal	Enablement
*Activity 2: Collaborative improvement meetings*		
Community of Practice met weekly to discuss challenges and devise solutions.	Problem solving	Enablement
Community of Practice members offered practical support to each other based on challenges discussed.	Social support (practical)	Enablement
Community of Practice members compared and discussed their own performance to that of their peers.	Social comparison	Modeling
Research staff gave Community of Practice members more decision-making power during the weekly meetings.	Restructuring the social environment	Environmental restructuring
*Activity 3: Real-time communications among members*		
Program staff created a WhatsApp group for support	Social support (practical)	Enablement
*Activity 4: Didactic education sessions*		
Community of Practice members identified gaps in their own knowledge and skills and requested appropriate education and training sessions.	Instruction on how to perform a behavior	Training, education
Community of Practice members gained self-efficacy and confidence to engage with communities as health workers.	Identity associated with changed behavior	Training, education
*Activity 5: Clinic-wide staff meetings*		
Community of Practice members were recognized for their contributions in the clinic.	Restructuring the social environment	Environmental restructuring
Community of Practice members received practical support from clinic staff.	Social support (practical)	Enablement
Community of Practice members shared problems with clinic staff to devise solutions.	Problem solving	Enablement

### Activity 1: Individual review of feedback reports

First, CHWs stated that review of feedback reports helped them improve the quality of contact investigation services. The research staff provided weekly performance reports to CoP members that included key indicators for the TB contact investigation cascade for each site. The weekly reports also included an itemized list of missing case record forms and the associated contacts for each individual CHW. CHWs could review their own individual reports to monitor their performance over time. One CHW explained:

“Those reports were very helpful in a way that it helped me figure out my weaknesses, where I had not done well. It would help me know the home visits I have and those I missed so I would know that I am demanded [responsible for] three home visits which was very helpful.” (CHW6, male, 43 years old)

Many respondents also suggested that reviewing and later referencing feedback reports served as “reminders” to complete any unfinished contact investigation activities. Thus, this theme mapped to the *self-monitoring of behavior* BCT and *enablement* Behavior Change Wheel function.

Next, respondents described how feedback reports enabled them to learn about their own individual performance as well as aggregate performance of all CHWs at the clinic. One CHW explained:

“For me the [feedback] dashboards were fine and they used to remind us, for example, when you forget and you did not do the clinical evaluation…how are you performing and how was the clinic also performing.” (CHW1, female, 26 years old)

Thus, through feedback reports, the CoP facilitated the *feedback on behavior* BCT and functioned through *enablement*.

CHWs suggested that feedback reports helped them gauge progress toward their objective of providing complete evaluation and linkage to treatment for all TB contacts. For example, one respondent explained:

“[Feedback reports] were good because they used to tell us what we should do, where we are delaying, what we are not doing well. Then we used to improve. They used to be good and in fact we should need them.” (CHW7, female, 30 years old)

By drawing attention to discrepancies between their activities and goals, CHWs emphasized that feedback reports helped them “get back on track.” This theme mapped to the *discrepancy between current behavior and goal* BCT and the *enablement* Behavior Change Wheel function.

### Activity 2: Collaborative improvement meetings

CHWs described their experiences participating in weekly collaborative improvement meetings where they shared their experiences performing contact investigation, reviewed feedback reports with peers, and solved problems together. One CHW explained:

“I had worked before [the CoP was established] and they [the clinic staff] used to not know the challenges which we had and thus I used to spend three months on a problem. There is no way you could get over it. When I got the meetings weekly, I would share my ideas and problems and thus get a solution at that time.” (CHW1, female, 26 years old)

This theme mapped to the *problem-solving* BCT and *enablement* intervention function.

CHWs described that the feedback report discussions enabled them to support each other to reach their goal of completing contact investigation. For example, one respondent explained:

“Whenever you would get a challenge you could discuss it with other [CoP] members and they would give you advice. Because everyone gets their experience in a different way. We came to know that if this patient is not comfortable with me, I can switch to another community health worker. And things are sorted. So it was really good.” (CHW3, female, 36 years old)

The CHWs recognized that each CoP member had particular experiences and skills that they could use to support each other. Thus, this theme mapped to the *social support (practical)* BCT and to the *enablement* intervention function.

CHWs also described that the collaborative improvement meetings enabled them to compare their own metrics against each other. The field notes indicated that the CoP discussed each member’s performance one-by-one. For example, weekly performance reports itemized and flagged incomplete contact investigation procedures as “missing forms.” One respondent explained:

“I would first rush and look at the pending [reports] I have, the missing forms I have. Then I look through and see my number and say ‘Ahh, I have two missing forms’ and others have ten, others have four. Then you would say ‘Why do you have four, why do you have ten?’ Then they would remind us of the missing people [household contacts] …Then when you go back, you call that home, you ask them what you were missing.” (CHW8, female, 45 years old)

Because CHWs directly compared their own performance to that of their peers during group audit-and-feedback, this theme mapped to the *social comparison* BCT and *modeling* intervention function.

Furthermore, CHWs described that the collaborative improvement meetings increased their professional autonomy. The field notes described instances when CHWs brainstormed solutions for problems they were facing and then presented these solutions to the research coordinator to enact change. In interviews, CHWs explained that they felt a shared sense of ownership of the CoP and this sense of ownership enhanced their decision-making power to propose and carry out innovations:

“They called us a team. Then they introduced to us what we were going to do and learned that it was going to majorly depend on our side, as the [CoP] team. Because assuming we got a problem and needed a solution, we had to sit together and see the way forward. So, majorly it was on our decision making... In our teaching, we usually get orders from above. You are told what to do. And it’s not from down to up. But this time it was from down to up. So it wasn’t expected that way. It was new.” (CHW2, male, 51 years old)

Many CHWs reported that having greater agency was motivating and enabled them to make meaningful changes to facilitate their work. This theme mapped to the *restructuring the social environment* BCT and *environmental restructuring* intervention function.

### Activity 3: Real-time communication among members

During weekly collaborative improvement meetings, CHWs concluded that having a system to communicate in real-time could facilitate timely support when problems arose in the field. The research team provided funding for and set up a WhatsApp messaging group to enable real-time communication between CoP members. The CHWs explained that having a WhatsApp group message gave them immediate access to a network of people should they experience a problem, especially in the field:

“If you needed some support, someone is there available for you to really support you with something that is challenging at the moment, which wasn’t there before…We developed a WhatsApp group. We used to communicate via phone calls and if one of the supervisors is not picking, [then] another is available. It really made some changes in the [communication] process.” (CHW3, female, 36 years old)

This theme mapped to the *social support (practical)* BCT and *enablement* function.

### Activity 4: Didactic education sessions

CoP members invited experienced clinicians to deliver didactic education sessions on topics of interest. For example, field notes suggested that the CoP enabled members to identify gaps in their knowledge and skills for screening TB in children. CHWs then requested a didactic session on TB in children:

“I didn’t know how to screen TB in children. But during the CoP, we got the skills through our doctor... He gave us other skills of screening TB in children… It has raised me from one level of just being a community health worker of general health care to a more skilled [one] in TB…I can screen a child for TB, and I can give a full session on TB in children and adults.” (CHW4, female, 45 years old)

The CHWs suggested that receiving educational sessions enabled them to improve their self-efficacy. This theme mapped to the *instruction on how to perform a behavior* BCT and *education* and *training* intervention functions.

Furthermore, CHWs shared that expanding their interpersonal and technical skills through the didactic sessions enabled them to work independently in the community as health workers, or *musawo*, similar to nurses and doctors. One respondent shared:

“[The CoP] taught me how to be patient with patients, it taught me how to be a humble person to patients and a loving person… sometimes it is not easy for a doctor or a nurse to go to a patient’s house but a CHW goes deep inside. And on the side of the patients, it makes them happy to see a musawo coming to his house sitting on that dirty chair, sitting on that dirty mat, it makes the patient happy.” (CHW5, female, 33 years old)

CHWs emphasized that the trainings enabled them to identify themselves as more skilled and compassionate health workers, mapping to the *identity associated with changed behavior* BCT and the *training* and *education* intervention functions.

### Activity 5: Clinic-wide staff meetings

During collaborative improvement meetings, CoP members discussed how to improve communication between CHWs and clinic staff to facilitate their work. Thus, the CoP began holding meetings between CHWs and Kampala Capital City Authority (KCCA) clinic staff, including the clinic in-charge, lab personnel, TB leader, and clinicians. These meetings provided an opportunity for CHWs to discuss their contributions with clinic staff. One CHW described:

“We were very much recognized by the KCCA people and I think they even appreciated the work that was done...Before they used to not recognize community health workers very much. They could minimize [our work] a bit. But by that time at least some change was there. They recognized what was done in the community because we used to even refer some other people for other problems, not only TB and HIV.” (CHW3, female, 36 years old)

Through clinic-wide staff meetings, clinic staff began recognizing CHWs’ work, improving their social standing within the clinic. This theme mapped to the *restructuring the social environment* BCT and *environmental restructuring* intervention function.

The CHWs explained that showcasing their successes to clinic staff also motivated clinic staff to support them when they faced barriers at the clinic. One respondent explained:

“They were all supportive, from sister in-charge to everyone, they were all supportive… in case I wanted anything, maybe from sister in-charge or lab or from a doctor or from a nurse, I could get it immediately…Because of the work I was doing. They saw that the work was good, they would make it easy for you.” (CHW5, female, 33 years old)

Thus, this theme mapped to the *social support (practical)* BCT and *enablement* intervention function.

These meetings also created opportunities for clinic staff and CHWs to problem solve together to improve TB care in the clinic and community. One CHW described:

“Another motivation was that we were able to communicate in our meetings… We had a chance to sit with our medical team of our facilities… so that anything beyond our capability was able to be solved because we had the medical personnel with us as we were discussing or sharing our problems and challenges.” (CHW2, male, 51 years old)

This theme mapped to the *problem-solving* BCT and *enablement* function.

## Discussion

A failure to implement, adapt, and sustain delivery of proven interventions is among the greatest barriers to control and elimination of TB [55], and CoPs offer an implementation strategy to achieve this goal through continuous group learning among healthcare workers. In this study, we used qualitative methods to catalog the activities that CHW participants of a CoP found to be most important to the CoP’s quality improvement efforts. We then categorized these activities using a well-established implementation framework and a linked taxonomy of behavior change. In so doing, we were able to specify the core and peripheral components and behavioral mechanisms of a working CoP. Our findings fill several important gaps in the literature related to how CoPs function, including how CoPs can operate in the context of a low-income country and how CoPs can be designed by researchers to improve delivery of evidence-based practices [10, 11]. This information may guide planning, implementation, and evaluation of CoPs in other similar settings [51, 56].

CHWs described five activities that were core to the workings of the CoP—two that were proposed by the research team to establish the CoP (i.e., review of feedback reports and collaborative improvement meetings) and three that were proposed and adopted by CoP members (i.e., real-time communications among members, didactic education sessions, and clinic-wide staff meetings). The collaborative improvement meetings and review of feedback reports provided a venue and opportunities for members to share challenges, exchange knowledge, engage in group problem solving, and benefit from social support. The three CoP-initiated activities facilitated social support in the field, restructured the social environment within and outside the clinic, and provided relevant education. CHWs described that being able to develop their own goals and activities was novel within their hierarchical work culture: “In our teaching, we usually get orders from above. You are told what to do. And it’s not from down to up. But this time it was from down to up.” Other studies of CoPs have also identified a contradiction between “getting orders from above” and being able to propose ideas “from down to up” [15, 16, 29, 57]. For example, one study of a CoP of healthcare workers to implement falls prevention strategies found that perceived lack of support from management in prioritizing the CoP’s goals and activities was a key barrier to their program [29]. These tensions between CoP goals and management goals should be further explored, especially within health settings that have well-established hierarchies, in order to ensure that CoPs retain their organic, bottom-up approach to learning.

Our study reinforces and builds upon a small existing literature describing the mechanisms of CoPs in low-income countries. Because other studies of CoPs have not used the same theoretical approaches to explicitly link activities to BCTs or intervention functions, our findings are not directly comparable. Nevertheless, the findings of prior studies do identify similar constructs as core to CoP functioning. For example, a study of a CoP including physicians, nurses, and pharmacists to improve HIV care in Namibia found that clinical knowledge and self-efficacy to deliver HIV services increased while professional isolation decreased after CoP implementation [32]. These findings suggest that this CoP may have functioned through the *instruction on how to perform a behavior* and *social support* BCTs, similar to our findings. Another study of a CoP including CHWs and traditional healers focused on Buruli ulcer care in Cameroon found that the CoP enabled CHWs to develop more autonomy in providing patient care, which led to improved social standing [31], consistent with the *restructuring the social environment* BCT. Similarly, CHWs in our study reported being motivated by the ways the CoP restructured the social environment and fostered increased autonomy and efficiency in solving problems. Taken together, these findings suggest that healthcare worker CoPs can facilitate delivery of high-quality care by enhancing members’ knowledge, self-efficacy, social support, and social status, which may be important motivators.

Our study provides preliminary insights into how CoPs can be designed, implemented, and supported by research teams in a way that enables CoP members to take ownership of the group. Our research team facilitated the establishment of a CHW CoP by integrating Wenger’s (2002)’s three elements of CoPs: (1) *domain*, (2) *community*, and (3) *practice* [34]. First, we invited CHWs to participate who had a common goal of improving contact investigation for TB (i.e., *domain*). We also suggested that CHWs meet weekly to promote *community* building and provided feedback reports to foster their *practice*. To encourage CHW ownership of the CoP, the chairperson of the weekly meetings rotated among all CHW participants. This structure enabled CHWs to develop their own group goals and norms after the research team initially implemented the CoP. Other research teams have also established CoPs by encouraging meetings to initially establish the group, and then gradually allowing members to take ownership of the CoP. For example, in a study of a CoP to improve the use of an evidence-based tool for assessing children’s mental health, the CoP was initially established by a facilitator [26]. In their initial CoP meeting, the facilitator described the purpose of CoPs and best practices for engaging with the community. In the following five meetings, the CoP members jointly outlined the agenda and led the sessions by describing issues that arose when implementing the evidence-based tool and providing each other with advice. Another study of a CoP to improve the quality of referral letters by general practitioners also initially established the group with the support of the research team [30]. The researchers recruited CoP members by inviting general practitioners that acknowledged the need for improving referral letters. Once in the group, the members defined the standards for quality of referral letters and set benchmarks for their goals. Despite these findings, there is a lack of clarity in the roles of the research team and project coordinators in facilitating and maintaining CoPs, and the extent to which the outcomes of the CoP are driven by the support of facilitators or by the CoP members themselves [11, 42]. For example, in the study of a CoP focused on improving the quality of referral letters, the research team identified that it was important to have a project coordinator regularly communicate with the CoP to maintain interest and motivation in the project [30]. Future studies of CoPs should more explicitly explore the extent to which the outcomes of the CoP were attributable to the CoP itself or the facilitation by the research team.

Our study has some limitations. Because the interviews were conducted by research staff, social desirability bias might have influenced CHWs to describe activities in an overly favorable way. Given the nature of qualitative research, our study may not be generalizable to CoPs with different members, goals, and contexts. Furthermore, our study included a single CoP with only eight members focused on TB contact investigation; additional studies of CoPs focused on other evidence-based practices will be needed to validate, modify, and/or refute our findings. Due to the small sample size, we were not able to identify differences in responses by CHW characteristics, such as age, gender, or years of experience. We interviewed CHWs five months after CoP initiation; thus, we could not assess long-term sustainability of the CoP or the extent to which it evolved over time. Although there was evidence that many aspects of the CoP were driven by CoP members, we were not able to explicitly differentiate how much of the success of the CoP was related to the facilitation and support from the research team. Lastly, due to the iterative nature of the development of the CoP and its activities, the quantitative data collected during the study period did not have adequate power to measure the effectiveness of the CoP. Instead, participants reported perceived improvements in contact investigation attributable to the CoP. Future studies would benefit from using a mixed-methods approach to assessing CoPs in order to quantitatively explore the effectiveness and behavioral mechanisms of CoPs.

Our study also had several strengths. First, our overall approach drew on key strategies to harness the power of theorizing in implementation science, including (1) approaching the empirical data in a theoretically informative way by analyzing the interviews using an abductive approach informed by the BCT Taxonomy and Behavior Change Wheel framework, (2) theorizing the dynamic relationships between the CoP and Ugandan context by identifying potential behavior change mechanisms, and (3) broadening the repertoire of major theoretical traditions by integrating classical social learning theories with implementation theories [43]. Furthermore, by combining inductive and deductive analytical approaches, we could identify the core components of the CoP from the perspectives of CHWs, while also standardizing our specification of the CoP to make it more transferrable to other similar settings, for replication, adaptation, and scale-up. Lastly, by triangulating our interview findings with field notes collected through observations, we aimed to mitigate social desirability bias and gain a comprehensive understanding of CoP activities.

## Conclusions

In summary, we identified the BCTs and intervention functions through which a CoP facilitated the delivery of high-quality TB care by CHWs in Uganda. Additional empirical studies are warranted to validate and/or modify these proposed core components to better understand how and under what conditions CoPs can be implemented to facilitate CHW-delivered health services. By using behavioral theory to better characterize CoPs, we hope that future CoPs can be appropriately adapted to maximize their effectiveness and sustainability.

## Supplementary Information

**Additional file 1.** Interview guide.

**Additional File 2.** Contextual factors that facilitated Community of Practice activities.

## Data Availability

The datasets used and/or analyzed during the current study are available from the corresponding author on reasonable request.

## Abbreviations

BCT: Behavior Change Technique
CHW: Community health worker
CoP: Community of Practice
COREQ: Consolidated Criteria for Reporting Qualitative Research
NTLP: National Tuberculosis and Leprosy Program
TB: Tuberculosis
TIDieR: Template for Intervention Description and Replication
