# Supplementary material for: Core components of a Community of Practice to improve community health worker performance: a qualitative study
Source: Implement Sci Commun. 2022 Mar 10;3:27. doi: 10.1186/s43058-022-00279-1 (PMC8908651; doi:10.1186/s43058-022-00279-1)
Supplement: Supplementary file 1 — Additional file 1. Interview guide. [file 43058_2022_279_MOESM1_ESM.docx]

**Additional File 1. Interview guide**

*Domain 1: Perception of role*

1. Looking at your current job working as a community health worker following a Community of Practice approach on the pilot study, is the job what you expected?

*Probes*:

How does it make you feel?

Does it make you excited?

1. In our previous discussion, we talked about what it means to be a community health worker, has that changed over time?

*Probes*:

What is it really like being a community health worker?

1. What has kept you encouraged (motivated) to continue doing community health worker activities?

*Probes*:

What parts of your work give you motivation?

1. In the last six months, has being a community health worker changed you in any way?

*Probes*:

How so?

How has being a CHW affected your life?

1. Which aspects of your job do you consider to be the most important? (e.g. collecting good quality sputum, delivering Xpert results to clients, doing HIV tests, etc.)

*Domain 2: Experiences during the Community of Practice pilot study*

1. What have been your experiences during the pilot study working with other departments at your clinic?
2. Has your experience working with the pilot changed your relationship with others at the clinic?
3. How did you find support for your work from others at your clinic?
4. Can you tell me about a time one of your fellow CHWs helped you?

*Probes*:

How did they help?

How did you ask them for help?

1. Do you look up to any of your colleagues? If yes, who and why?

*Domain 3: Feedback reports*

1. How do you find the dashboards?

*Probe:*

Which part(s) of the report do you find most useful?

1. When you are given a weekly report, what do you look at first?

*Domain 4: Thank you and conclusion*

1. Is there anything else you want to tell me about being a CHW?
2. Is there any question I didn’t ask that I should have asked
