# Supplementary material for: Core components of a Community of Practice to improve community health worker performance: a qualitative study
Source: Implement Sci Commun. 2022 Mar 10;3:27. doi: 10.1186/s43058-022-00279-1 (PMC8908651; doi:10.1186/s43058-022-00279-1)
Supplement: Supplementary file 2 — Additional File 2. Contextual factors that facilitated Community of Practice activities. [file 43058_2022_279_MOESM2_ESM.docx]

**Additional File 2. Contextual factors that facilitated Community of Practice activities**

| **Community of Practice Activity** | **Contextual Factor(s)** | **Quotation** |
| --- | --- | --- |
| Individual review of feedback reports | The tablet reader and electronic mode of delivering the feedback reports, once adopted, provided a more timely and readily accessible way for Community of Practice members to receive feedback. | “Because we were able to get our reports without moving from our work places, because whatever we were doing was directly reported, the paper [report] was not so much needed. We were able to give in our reports as soon as we have finished our client, a report was directly sent to the server so there was an easy follow [up] assuming there was something which did not go well. And at least there was easy communication to correct the problem or mistake instead of waiting or calling, you were able to get out of that dilemma as soon as it happened.” |
| Collaborative improvement meetings | The electronic feedback reports on the tablets enabled Community of Practice members to easily review each other's reports for discussion during the meetings. | “So you could be talking [in the CoP meetings] and the then the person there could follow whatever was being discussed and then scroll in time... You know if you are sharing a soft [electronic] copy it is easier. They could share it with these people through email so anyone could have it soon and they would love it.” |
| Real-time communications among members | Access to a mobile data plan and supportive supervisors facilitated communication among Community of Practice members and supervisors in the field. | “First of all, our supervisors used to help us in all areas where we could get challenges. They were always readily available. You could call them whether you were in a home visit and they could say no, you can do this or that, so they were always there for us. They could also provide us with timely transport to go to those home visits. We were very well facilitated. So it could make work easy for me to plan with a patient and visit as soon as possible.” |
| Didactic education sessions | None | N/A |
| Clinic-wide staff meetings | Supportive supervisors advocated for Community of Practice members at the clinic. | “You would call [CoP supervisors] if something is not done [at the clinic] and they would come to the [Health Center] and we would have a meeting on the issues.” |
